# Prioritizing Equity in Antimicrobial Stewardship Efforts (EASE): a framework for infectious diseases clinicians

**DOI:** 10.1017/ash.2024.69

**Published:** 2024-05-03

**Authors:** Jacinda C. Abdul-Mutakabbir, Karen K. Tan, Candace L. Johnson, Caitlin L. McGrath, Danielle M. Zerr, Jasmine R. Marcelin

**Affiliations:** 1 Division of Clinical Pharmacy, Skaggs School of Pharmacy and Pharmaceutical Sciences, University of California San Diego, La Jolla, CA 92093, USA; 2 Division of Black Diaspora and African American Studies, University of California San Diego, La Jolla, CA 92093, USA; 3 Loma Linda University Medical Center, Department of Pharmacy, Loma Linda, CA, USA; 4 Loma Linda University School of Pharmacy, Loma Linda, CA 92374, USA; 5 Department of Pediatrics, Division of Pediatric Infectious Diseases, Columbia University Irving Medical Center, New York, NY 10032, USA; 6 Department of Infection Prevention and Control, NewYork-Presbyterian Hospital, New York, NY 10032, USA; 7 Department of Pediatrics, Division of Pediatric Infectious Diseases, University of Washington School of Medicine, Seattle, WA 98105, USA; 8 Division of Infectious Diseases, University of Nebraska Medical Center, Omaha, NE 68198, USA

## Abstract

Health equity gaps persist across minoritized groups due to systems of oppression affecting health-related social needs such as access to transportation, education and literacy, or food and housing security. Consequently, disparities in the prevalence of multidrug-resistant infections, infectious disease outcomes, and inappropriate antimicrobial use have been reported across minoritized populations. The Joint Commission and Centers for Medicare and Medicaid Services (CMS) have formally acknowledged the importance of integrating health equity-focused initiatives into existing hospital quality improvement (QI) programs. Here, we review documented disparities in antimicrobial stewardship and offer a framework, derived from components of existing health equity and QI tools, to guide clinicians in prioritizing equity in antimicrobial stewardship efforts (EASE).

## Introduction

The United States has become increasingly diverse, with a dramatic rise in the number of racially and ethnically minoritized groups over the past decade.^1^ Concurrently, more people are disclosing and acknowledging intersectional identities including sexual and gender minorities and people with disabilities, among others.^2,3^ Despite this shift in national demographics, healthcare disparities remain prevalent for individuals from minoritized populations.^4,5^ This was showcased during the coronavirus disease 2019 (COVID-19) pandemic, as people from racially and ethnically minoritized groups, sexual and gender minority groups, and individuals residing in areas of low socioeconomic status (SES) were shown to be at increased risk of disease severity and less likely to receive access to necessary therapeutics.^6,7^ Furthermore, multiple authors also report disparities in microbial diagnoses and the appropriateness of the antimicrobials prescribed to minoritized individuals.^8–11^ The confluence of multiple, overlapping systems of oppression (including, but not limited to racism, homophobia, and ableism) impacting health-related social needs (e.g., education, employment, access to health services, etc.) have been attributed as root causes of the reported inequities.^12–14^

Recognizing the critical importance of addressing health equity to enhance patient care, the Centers for Medicare and Medicaid Services (CMS) have recently published their updated Framework for Health Equity 2022-2032.^15^ This comprehensive framework outlines five key health equity priorities: (1) expanding the collection, reporting, and analysis of standardized data; (2) assessing causes of disparities within CMS Programs, and addressing inequities in policies and operating to close gaps; (3) building the capacity of healthcare organizations and the workforce to reduce healthcare disparities; (4) advancing language access, health literacy, and the provision of culturally tailored services; and (5) increasing all forms of accessibility to healthcare services and coverage. In alignment with these priorities, the Joint Commission has also released new requirements, effective January 1, 2023. These requirements include the establishment of a dedicated leadership team, the patient assessment of health-related social needs, and the stratification of quality and safety data using sociodemographic characteristics.^14^

Although the standards and guidance provided by the Joint Commission and CMS are helpful initial resources to use in identifying and addressing health disparities, there is a need for a tailored tool to guide clinicians through the process of mitigating antimicrobial stewardship (AS) related inequities. Here, we review AS disparities and provide a framework for clinicians to use in prioritizing equity in AS efforts (EASE).

### Overview of antimicrobial stewardship-associated inequities

The Centers for Disease Control and Prevention (CDC) estimates that more than 2.8 million multidrug-resistant (MDR) infections occur annually, contributing to increased infection-related mortality and morbidity.^16^ AS-related interventions have been shown to play a vital role in optimizing antimicrobial use by escalating/de-escalating therapy and monitoring for safety and potential adverse effects related to antimicrobial usage.^17^ Nonetheless, there is an ever-present opportunity for re-examining equity in institutional AS activities as various studies have described the association between health-related social needs and deleterious infectious disease (ID)-related outcomes observed across minoritized groups.^18^

Individuals who reside in areas of low SES reportedly have less access to adequate education opportunities.^19,20^ Thus, these groups are more likely to have basic or below basic health literacy when compared to those individuals residing in areas of higher SES.^19,20^ Several investigators have described the potential consequences that may stem from the decreased health literacy recognized across low SES groups.^10,21^ Notably, Mcleod et al conducted a retrospective observational study that focused on identifying racial and social vulnerability differences in the diagnosis and management of acute cystitis treated in the emergency department (ED) and two urgent care centers associated with Loma Linda University Health.^10^ In the study, women belonging to racially and ethnically minoritized groups and residing in the areas of highest vulnerability (low SES), were more likely to be diagnosed as having acute cystitis and were significantly younger than their non-Hispanic White counterparts diagnosed with acute cystitis (median age 47 vs 67 years). Noting the low health literacy rates across highly vulnerable areas, the authors suggest that tailored educational tools for the uncovered disadvantaged group may be a viable intervention for addressing the observed inequity.^10^ It is also important to note that 79/114 (69%) of the patients identified as Hispanic/Latino. While the authors do not specifically state this point, given limitations in translated health resources, language barriers may also have been a limitation to patient health literacy.^10,22^

While not specific to AS, Kristensen et al describe disparities in the adherence to methicillin-resistant *Staphylococcus aureus* posttreatment follow-up and successful decolonization based on SES factors.^21^ The authors state that the factors associated with success were individuals with higher education, early retirees, and those living in urban municipalities.^21^ Given the linkage of higher education to increased health literacy, the authors hypothesize that those individuals with lower education (literacy) levels, were less likely to be decolonized potentially due to their difficulties related to understanding health information and communicating with clinicians.^21^ Overall, these studies highlight the connection between education/literacy (or lack thereof), social vulnerability, and exacerbated disparities in ID-related diagnoses and management.

Social vulnerability (also described as deprivation) has also been associated with a limitation in access to services such as outpatient antimicrobial therapy (OPAT) even in countries with universal health care.^23^ This is detrimental as OPAT can be utilized as a cost-effective approach to early discharge or to avoid hospitalization for patients requiring intravenous antimicrobials. In a UK-based study, Sumpter et al reported social disparities in OPAT referrals for cellulitis, indicating that individuals with lower SES scores were less likely to receive OPAT referrals. Using the Scottish Index of Multiple Deprivation—an area-based measure of relative deprivation that takes into account income, employment, education, and access to health services—the authors found the individuals with the lowest scores (most deprived) were less likely to receive an OPAT referral when compared to more affluent patients.^23^ Additionally, women were almost a third less likely to be referred for OPAT compared to men, irrespective of adjustments for age, number of co-morbidities, admissions, and length of stay.^23^

Furthermore, insurance status has also been utilized as a marker of patient vulnerability to poor ID outcomes.^24^ Low-income adults insured by Medicaid had a higher *Clostridioides difficile* infection (CDI) incidence when compared to those who were commercially insured. Additionally, uninsured patients are less likely to be vaccinated against preventable diseases, which can also exacerbate negative outcomes.^25^

The inequities described could have dire consequences which may include the continued propagation of MDR organisms, which will extend beyond bacterial infections.^26^ This is evidenced by a study conducted by Grant et al where the authors reported racial differences in candidemia in an academic institution located in a highly vulnerable area.^26^ The authors observed higher rates of candidemia infections in racially and ethnically minoritized patients, predominantly Hispanic/Latino patients when compared to non-racially and ethnically minoritized patients.^26^ The investigators also reported disproportionately higher rates of *Candida parapsilosis* infections in racially and ethnically minoritized patients. This increase is concerning considering that *C. parapsilosis* azole resistance is globally increasing, reflected in the authors’ note that 27% of their study’s isolates were azole-resistant.^26^

In addition to the influence of health-related social needs inequities on ID outcomes, clinician biases may contribute to observed disparities across minoritized groups. A retrospective cohort study conducted by Goyal et al reported that racially and ethnically minoritized patients, including non-Hispanic Black and Hispanic/Latino individuals, were less likely to receive antibiotics for a viral infection compared to their non-racially and ethnically minoritized counterparts.^27^ Although this outcome was in line with AS goals, the authors attributed this prescribing difference to parental expectations and implicit prescriber bias, where non-Hispanic White children were perceived as more severely ill than their minoritized peers.^27^ These unconscious biases become problematic over the lifetime for racially and ethnically minortized patients. This is highlighted through studies conducted in adult patients where individuals from minoritized groups were less likely to be prescribed antimicrobials for the treatment of severe infections, resulting in poorer outcomes.^8,26,28^ This demonstrates the necessity of identifying clinician biases and addressing behaviors that have the potential to widen health equity gaps.

**Figure 1.**
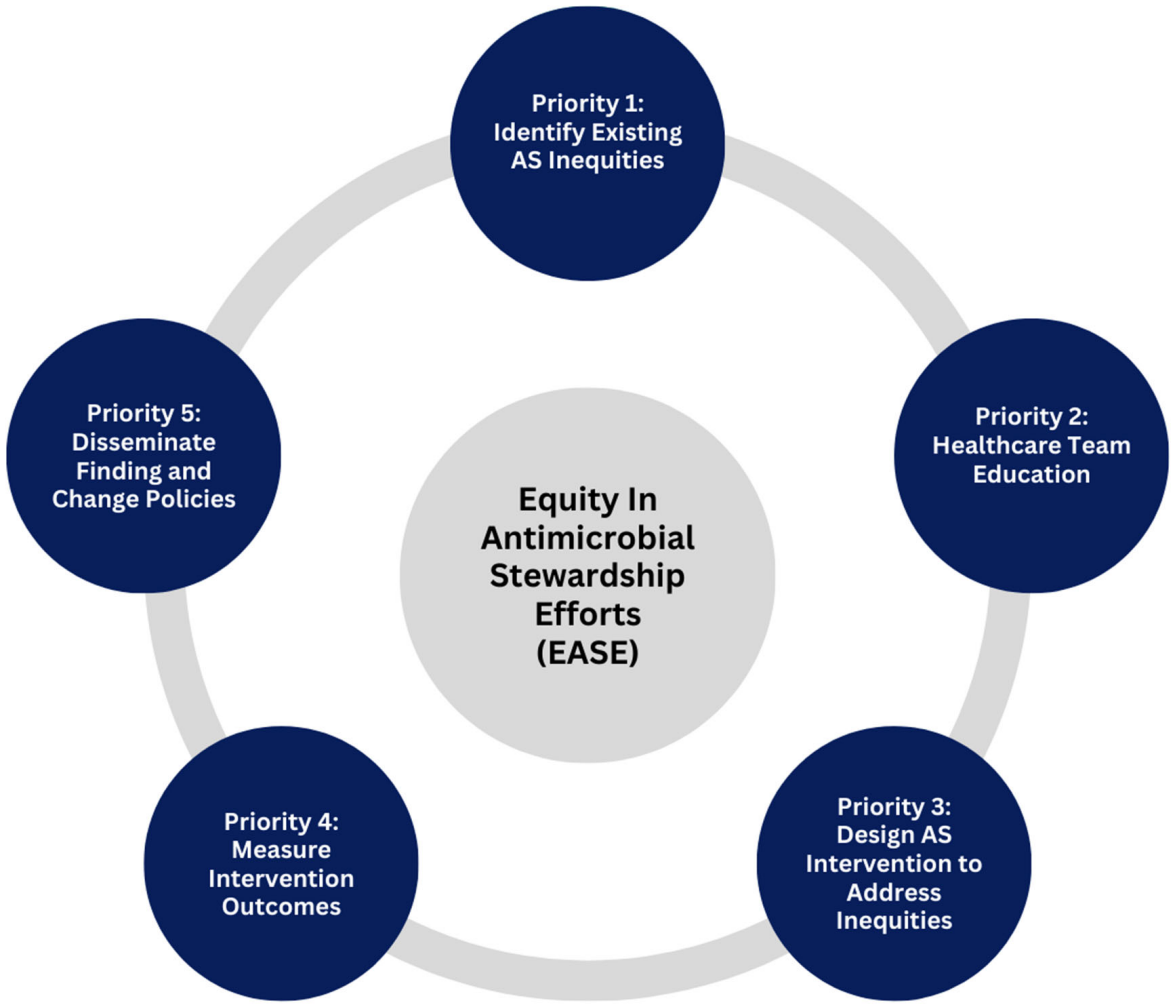
Prioritizing Equity in Antimicrobial Stewardship Efforts (EASE) framework.

Ultimately, these studies underscore the importance of clinician education on structural barriers, systemic bias, and health-related social needs and their potential influence on ID outcomes. Moreover, they highlight the need for the development and implementation of a tool that guides sustainable interventions designed to prioritize equity throughout AS initiatives. We developed the Prioritizing Equity in Antimicrobial Stewardsdhip Efforts (EASE) framework (Figure 1) to address this need.

### Prioritizing Equity in Antimicrobial Stewardship Efforts (EASE)

Although most AS interventions include quality improvement (QI) components focused on optimizing antimicrobial prescribing, there remains a significant gap in the integration of variables that measure health-related social needs inequities and areas for intervention.^11^ Reimagining AS through an equity lens, as described by Cichon et al, involves enhancing access to AS, reducing prescribing disparities, launching targeted public health campaigns, and ensuring representation in decision-making.^18^

Globally and nationally, healthcare governing agencies such as the World Health Organization (WHO) and the CDC have declared health an essential human right.^12,29^ Thus, the conceptual framework for action on the social determinants of health (CSDH) and the Healthy People 2030 framework developed by WHO and the CDC, respectively are well positioned as helpful aids for clinicians to utilize in systematically identifying health-related social needs.^12,29^ In addition to need identification, it is also imperative for clinicians to have practical and tangible priorities and examples to guide potential interventions to mitigate the observed inequities; this is highlighted by the aforementioned CMS framework for health equity.^15^

Our framework proposed in Figure 1 incorporates applicable aspects of the WHO, CDC, and CMS frameworks—in context with previously described functions of AS—to distinguish practical priorities to consider for integrating equity within AS-focused activities.

### The EASE framework as a quality improvement process

Recognizing and designing interventions that address inequities will likely require multiple evaluations and iterations, like the plan-do-study-act cycle in a QI process.^30^ We have designed this framework to be utilized in this manner. Of note, the elements included in this framework are not meant to be exhaustive but to provide a starting point for future interventions. The priorities are in place to serve as checkpoints for the fulfillment of the key equity-focused areas described by the Joint Commission and CMS.^14,15^ Additionally, we provide strategies to consider when addressing each priority and we provide an expanded discussion on examples from existing literature that showcase the execution of the proposed priorities.

### Priority 1: Identify existing AS inequities

#### Strategy

Identifying the existing AS inequities, through data evaluation, is a critical first step in rectifying disparities, as emphasized by the Joint Commission.^14^ The task of where to begin this identification can be daunting, therefore, selecting one area of focus could be beneficial in reducing the perceived burden of the priority. Given the ability to collect race, ethnicity, and language (REaL) data directly from patients and record them in the hospital’s electronic medical record, these variables may be the most feasible to use as a starting point. The area of focus may vary by disease state or antimicrobial usage, however the disaggregation of the data by minoritized status (race and ethnicity, native language, etc.) or health-related social needs is imperative to identify the existing gaps.

#### Example and explanation

*C. difficile* infection (CDI) is often utilized as a surrogate marker for AS proficiency and may serve as a viable disease to begin with in uncovering inequities. Lee et al conducted a study focused on identifying racial and ethnic differences in CDI; thus, there was an intentional prioritization in the disaggregation of the racial/ethnic groups of included participants.^31^ Through stratification of the data, the authors found that patients who identified as Hispanic/Latino were more likely to be younger and to have extended ICU admission compared to non-Hispanic/Latino White patients diagnosed as having CDI. Further, they detected that racially and ethnically minoritized patients had 1.63 increased odds of presenting severe/fulminant CDI when compared to the non-racially and ethnically minoritized patient cohort with 10% of the total effect mediated by a preexisting chronic kidney disease (CKD) diagnosis.^31^ By selecting a clear starting point and disaggregating the data, the investigators were able to uncover CDI inequities and identify optimal patient populations where CDI clinical pathways can be designed to better provide care.^31^

Integrating vulnerability indices that measure health-related social needs may help uncover additional disparities and identify the optimal placement for planned interventions.^32,33^ Using the CDC Social Vulnerability Index (SVI)—which incorporates individual zip codes to measure deprivation based on 16 factors including race and ethnicity status, SES, housing, transportation, and household characteristics—investigators reported that patients in the lowest SES quartile had a higher prevalence of MDR Enterobacterales infections relative to the highest SES quartile.^8^ Using the SES sub-score, available via the CDC SVI tool, the investigators were able to delineate the study participants into four equal-sized quartiles: high, medium-high, medium-low, and low SES. By disaggregating the data, the authors were able to uncover the described MDR disparity and are now able to tailor future interventions and education for patients based on their findings.

### Priority 2: Healthcare team education

#### Strategy

There is a notable variation in the understanding of antimicrobial use and harms among different clinical disciplines and practice settings, therefore, providing directed education on the discovered AS equity gaps is critical. The education provided may resemble ID team members directly speaking with non-ID colleagues, the dissemination of the data in hospital-wide presentation format, or as easily consumable handouts. The teachings should highlight the inequity unveiled by the data and describe the relationship between ID-related inequities and other patient co-morbidities, where applicable. Additionally, the education should inform the clinicians, including those who are a part of the ID team, about implicit/explicit biases and describe their impact on the health disparities uncovered. The educational materials should also incorporate resources for continued learning on the topic to promote sustainable change. Echoing the Joint Commission recommendations, this should also be connected to departmental and institutional objectives and key results to further engage buy-in from prescribers and leaders.^14^

#### Example and explanation

In a study conducted by McGrath et al, investigators aimed to identify inequities for those in minoritized racial, ethnic, and language groups in pediatric central line-associated bloodstream infections (CLABSIs) and then develop a tailored intervention to mitigate the observed inequities.^34^ With the clear study objectives defined, the investigators disaggregated race and ethnicity and language of care for patients diagnosed as having CLABSI and for patients with central lines.^34^ There were higher rates of infection in patients who spoke a language other than English, and in those self-identifying as Black or African American.^34^ After identifying the disparity the investigators employed interventions that included prioritizing the communication of the findings with the hospital staff and engaging in multidisciplinary efforts to understand potential drivers for the uncovered disparities. They also aligned their interventions with the anti-racism and bias in healthcare education efforts that were ongoing at their organization during the study period to ensure that everyone involved would have the foundational tools necessary for long-lasting behavioral change.^34^

### Priority 3: Design AS intervention to address inequities

#### Strategy

Following targeted education, identifying decision-makers for the development of an intervention designed to address the exposed inequities is necessary. First, an advisory board with essential personnel, including a multidisciplinary AS team (ex: MD/DO, PharmD, nurse, microbiologist), patient representatives, and health equity staff (if available) should be assembled to discuss the results of the study and the inequities identified. Institutional social workers—who are trained to consider how the system in place has enacted policies and programs that disenfranchise certain communities—are also a strong asset in helping to bridge gaps in health equity and can be key additions to the team, especially in the absence of designated health equity staff.^35^ It is imperative to select representative advisors from diverse backgrounds, as the differences in perspectives allow for multiple minoritized identities to be considered and accounted for. Additionally, the collaboration and involvement of the hospital executives in the advisory board should be prioritized as their investment is critical to the success and sustainability of in the intervention.^14^ Led by an identified champion—as advised by the Joint Commission standards—the advisory board should identify a specific inequity to address and design an intervention that leverages the tools that the institution has at its disposal.^14^ This should be undertaken in a practical, stepwise fashion in collaboration with (rather than competing against) existing QI projects.^30^ Utilizing SMARTIE goals described by the Management Center (Specific, Measurable, Attainable, Relevant, Time-bound, Inclusive, and Equitable), teams can identify exactly *how* they could reach the next step in transformation using available resources.^36^

#### Example and explanation

Returning to the McGrath et al. study, following the identification of disparities in CLABSI events (Priority 1 in the (EASE) framework) the investigators describe providing education to hospital staff on the observed inequities^34^ (Priority 2). The authors discovered that fewer central line observations were being done in patients identifying as Black or African American and those who spoke a language other than English. They developed a tailored intervention that involved tracking line observations to ensure that minoritized patient groups were being observed with a frequency commensurate with the line days they were contributing (Priority 3). The authors then re-evaluated the outcomes in the patients to assess whether this intervention and others addressed the recognized inequities (Priority 4). The authors reported that following the intervention lower CLABSI rates were observed in Black or African American patients and in those that spoke a language other than English (intervention-focused groups).

### Priority 4: Measure intervention outcomes

#### Strategy

To ensure that the intervention is optimized to provide the best results, measurable outcomes should be determined to track the success or areas for improvement following the implementation of the initiative.^14^ Adoption of a quasi-experimental research design may aid in the ease of observing changes following the implementation of an intervention, and measurable outcomes may include a reduction in infection-related mortality or a decline in hospital length of stay, as these may both align with community commitments and hospital objectives and key results.^37,38^ The results from the measured outcomes should be used to guide any modifications necessary to better optimize the AS intervention. Also, if the outcomes denote a significant lack in progress, then it is important to re-engage key decision-makers to assess and determine additional actions for the success of the intervention.

#### Example and explanation

In a retrospective study, Lora et al assessed the feasibility of a COVID-19 monoclonal antibody (mAb) infusion site established in the ED of a safety-net hospital located in reaching vulnerable communities.^28^ The authors state that the program was placed in the ED as a means of reducing barriers for minoritized individuals to access COVID-19 mAb therapeutics, as minoritized patients were noted as having lower access and uptake for the therapeutics before designing the intervention.^28^ Following the establishment of the program, the authors disaggregated the data by zip code and race and ethnicity to determine the utilization of the mAbs at the institution.^28^

Using the zip codes and the CDC SVI the authors defined the zip codes as low, medium, or high vulnerability to COVID-19. Ultimately, the investigators found that their program was effective in serving highly vulnerable communities, with 64% of the mAb doses being provided to patients who had a high SVI score.^28^ Additionally, through the disaggregation of the race and ethnicity data, the authors noted that 77.2% of the doses were given to patients who identified as racially and ethnically minoritized.^28^ By developing a tailored program based on a recognized inequity (differences in the number of racially and ethnically minoritized and socially vulnerable patients that receive mAbs) and designing a tailored intervention to increase the use of mAbs in these populations, the authors were able to measure related outcomes.^28^ Ultimately, they were able to demonstrate the feasibility of their intervention and validate the success of their intervention in ensuring the equitable distribution of necessary therapeutics,^28^ thus serving as a model that can potentially be adapted for other stewardship initiatives within those communities of focus.

### Priority 5: Disseminate findings and change policies

#### Strategy

The widespread dissemination of the findings and strategies utilized in the intervention is important for the continued learning and growth of equity-focused AS, universally. Promoting advancements in this area can include a variety of activities such as including equity-focused sessions in scientific conference programming or soliciting research articles that describe AS inequities and/or describe equity-focused AS interventions. The consistent production and amplification of data that displays AS disparities will further emphasize the need for public attention and governmental funding to support continued research and advocacy for health equity.

#### Example and explanation

Several professional organizations have prioritized the solicitation of equity-focused articles for their respective journals. The Society of Healthcare Epidemiology of American (SHEA) journals, Antimicrobial Stewardship & Healthcare Epidemiology (ASHE) and Infection Control and Hospital Epidemiology (ICHE), specifically welcome and prioritize publications with a focus on equity-grounded interventions in AS and infection control.^39^ Furthermore, health equity-focused research tracks have been integrated into the programming of organizational annual conferences. In addition to presenting findings and experiences, ID professionals must advocate for equitable policy reform. ID organizations, such as SHEA and the Infectious Diseases Society of America (IDSA), provide opportunities and resources for ID professionals to engage in advocacy campaigns designed to ensure equity across the AS continuum.^40,41^

### Conclusion

Narrowing equity gaps in AS will require consistent education and innovation. Acknowledging the role of health-related social needs and clinician bias on AS disparities is paramount, as is a commitment to mitigating the widened equity gaps. The EASE framework, developed to aid ID clinicians in integrating the equity-focused Joint Commission and CMS standards into AS activities, provides several long-term priorities to ensure that each patient receives optimal care—irrespective of race, ethnicity, religion, sexuality, gender, ability status, or any other identity. Although each priority may not be addressed in a linear time line and is not exhaustive enough to account for the many iterations that these interventions will undergo, they provide a helpful starting point for promoting long-lasting, equitable change, in Antimicrobial Stewardship.
